# Cyclosporine-assisted adipose-derived mesenchymal stem cell therapy to mitigate acute kidney ischemia–reperfusion injury

**DOI:** 10.1186/scrt212

**Published:** 2013-05-31

**Authors:** Yen-Ta Chen, Chih-Chau Yang, Yen-Yi Zhen, Christopher Glenn Wallace, Jenq-Lin Yang, Cheuk-Kwan Sun, Tzu-Hsien Tsai, Jiunn-Jye Sheu, Sarah Chua, Chia-Lo Chang, Chung-Lung Cho, Steve Leu, Hon-Kan Yip

**Affiliations:** 1 Division of Urology, Department of Surgery, Kaohsiung Chang Gung Memorial Hospital and Chang Gung University College of Medicine, 123 Dapi Road, Niaosong Dist., Kaohsiung, Taiwan; 2 Division of Nephrology, Department of Internal Medicine, Kaohsiung Chang Gung Memorial Hospital and Chang Gung University College of Medicine, 123 Dapi Road, Niaosong Dist., Kaohsiung, Taiwan; 3 Division of Cardiology, Department of Internal Medicine, Kaohsiung Chang Gung Memorial Hospital and Chang Gung University College of Medicine, 123 Dapi Road, Niaosong Dist., Kaohsiung, Taiwan; 4 Department of Plastic Surgery, University Hospital of South Manchester, Southmoor Road, Manchester, M23 9LT, UK; 5 Center for Translational Research in Biomedical Sciences, Kaohsiung Chang Gung Memorial Hospital and Chang Gung University College of Medicine, 123 Dapi Road, Niaosong Dist., Kaohsiung, Taiwan; 6 Department of Emergency Medicine, E-DA Hospital, I-Shou University, No.1, Yida Road, Jiaosu Village, Yanchao District, Kaohsiung, Taiwan; 7 Division of thoracic and Cardiovascular Surgery, Department of Surgery, Kaohsiung Chang Gung Memorial Hospital and Chang Gung University College of Medicine, 123 Dapi Road, Niaosong Dist., Kaohsiung, Taiwan; 8 Division of Colorectal Surgery, Department of Surgery, Kaohsiung Chang Gung Memorial Hospital and Chang Gung University College of Medicine, 123 Dapi Road, Niaosong Dist., Kaohsiung, Taiwan; 9 Department of Biological Sciences, National Sun Yat-Sen University, 70, Lien-Hai Road, Kaohsiung, Taiwan

## Abstract

**Introduction:**

This study tested the hypothesis that cyclosporine (CsA)-supported syngeneic adipose-derived mesenchymal stem cell (ADMSC) therapy offered superior attenuation of acute ischemia–reperfusion (IR) kidney injury to either therapy alone.

**Methods:**

Adult Sprague–Dawley rats (*n* = 40) were equally divided into group 1 (sham controls), group 2 (IR injury), group 3 (IR + CsA (20 mg/kg at 1 and 24 hours after procedure)), group 4 (syngeneic ADMSC (1.2×10^6^) at 1, 6 and 24 hours after procedure), and group 5 (IR + CsA-ADMSC).

**Results:**

By 72 hours after the IR procedure, the creatinine level and the ratio of urine protein to creatinine were highest in group 2 and lowest in group 1, and significantly higher in groups 3 and 4 than in group 5 (all *P* <0.05 for inter-group comparisons), but showed no differences between groups 3 and 4 (*P* >0.05). The inflammatory biomarkers at mRNA (matrix metalloproteinase-9, RANTES, TNF-α), protein (TNF-α, NF-κB, intercellular adhesion molecule-1, platelet-derived growth factor), and cellular (CD68^+^) levels of IR kidney showed a similar pattern compared with that of creatinine in all groups (all *P* <0.05 for inter-group comparisons). The protein expressions of oxidative stress (oxidized protein), reactive oxygen species (NADPH oxidases NOX-1, NOX-2), apoptosis (Bcl-2–associated X protein, caspase-3 and poly(ADP-ribose) polymerase) and DNA damage (phosphorylated H2A histone family member X-positive, proliferating cell nuclear antigen-positive cells) markers exhibited a pattern similar to that of inflammatory mediators amongst all groups (all *P* <0.05 for inter-group comparisons). Expressions of antioxidant biomarkers at cellular (glutathione peroxidase, glutathione reductase, heme oxygenase-1 (HO-1)) and protein (NADPH dehydrogenase (quinone)-1, HO-1, endothelial nitric oxide synthase) levels, and endothelial progenitor cell markers (C-X-C chemokine receptor type 4-positive, stromal cell-derived factor-1α-positive) were lowest in groups 1 and 2, higher in groups 3 and 4, and highest in group 5 (all *P* <0.05 for inter-group comparisons).

**Conclusion:**

Combination therapy using CsA plus ADMSCs offers improved protection against acute IR kidney injury.

## Introduction

The kidney and its vital functions are vulnerable to damage by a variety of disease processes given its frequent exposure to reactive oxygen species (ROS) and toxic organic substances and its sensitivity to hemodynamic instability such as following hypotensive shock [1-6]. Of these processes [1-4,7], acute kidney injury caused by ischemic and/or ischemia–reperfusion (IR) injury [2,4,5] remains one of the most important problems to be solved for daily clinical practice [8-10]. Acute kidney injury lacks effective management and yet is responsible for high levels of inpatient morbidity and mortality [1-3,8-10]. An effective and safe treatment for acute kidney injury is therefore important and urgent for clinicians and scientists alike.

The underlying mechanism of acute organ IR injury mainly involves an ROS burst during reperfusion of ischemic tissues that can trigger opening of the mitochondrial permeability transition (MPT) pore, mitochondrial depolarization, decreased ATP synthesis and increased ROS production [11-14]. ROS generation stimulates pro-apoptotic mediators, inflammatory cytokines, further oxidative stress and exacerbation of inflammation [11-14]. The MPT pore comprises cyclophilin D, voltage-dependent anion channels and adenine nucleotide translocase [15-17]. Cyclosporine A (CsA), a cyclophilin D inhibitor, is well recognized to reduce ROS generation by inhibiting cyclophilin D action in the MPT pore [18-21]. Indeed, CsA administration limited myocardial infarct size [22] and protected organs against acute IR injury [21,23].

Moreover, numerous experimental studies [24,25] and clinical observational studies [26,27] have supported mesenchymal stem cell (MSC) therapy being a safe and promising modality for reversing ischemia-related organ dysfunction [24-27] and improving clinical outcome [26,27] mainly through angiogenic and paracrine effects. Furthermore, recent data have revealed that MSCs have intrinsic anti-inflammatory and immunomodulatory properties [28,29]. Importantly, MSC therapy reduced acute organ IR injury [5,6], including acute kidney IR injury [5]. Interestingly, an *in vitro* study has previously shown that human adipose tissue-derived MSCs facilitate the immunosuppressive effect of CsA on T lymphocytes through Jagged-1/Notch-related inhibition of NF-κB signaling [30]. However, further preclinical experimental study should be investigated to further confirm the safety the efficacy of this combination therapy prior to applying this strategic management for patients with acute kidney IR injury.

Given the above properties of CsA and MSCs, this study tested the hypothesis that CsA-supported adipose-derived mesenchymal stem cell (ADMSC) therapy might provide improved attenuation of acute kidney IR injury in a rat model.

## Methods

### Ethics

All animal experimental procedures were approved by the Institute of Animal Care and Use Committee at Chang Gung Memorial Hospital – Kaohsiung Medical Center (Affidavit of Approval of Animal Use Protocol No. 2008121108) and were performed in accordance with the Guide for the Care and Use of Laboratory Animals (NIH Publication No. 85–23, National Academy Press, Washington, DC, USA, revised 1996).

### Animal groups and isolation of adipose tissue for culture of adipose-derived mesenchymal stem cells

Pathogen-free, adult male Sprague–Dawley rats (*n* = 40) weighing 320 to350 g (Charles River Technology, BioLASCO Taiwan Co. Ltd, Taiwan) were randomized and equally divided into: group 1, sham controls (*n* = 8); group 2, acute kidney IR injury with DMEM medium (500 μl) without fetal bovine serum, at 1, 6 and 24 hours after the IR procedure (*n* = 8); group 3, acute kidney IR injury + CsA (20.0 mg/kg intravenously at 1 and 24 hours after the IR procedure (*n* = 8); group 4, acute kidney IR injury + syngeneic ADMSC (1.2 × 10^6^) at 1, 6, and 24 hours after the IR procedure (*n* = 8); and group 5, acute kidney IR injury + CsA + autologous ADMSC (1.2 × 10^6^) at 1, 6, and 24 hours after the IR procedure (*n* = 8). CsA and syngeneic ADMSC dosages were chosen according to our previous studies with minor modifications [5,21,23]. Additionally, the choice of time points for ADMSC administration at 1, 6, and 24 hours after the IR procedure was based on our recent study which demonstrated that ADMSC administration at these time points after acute rat kidney IR injury through penile venous transfusion markedly attenuated acute IR-induced kidney injury [5,21,23]. Besides, these time points were initially chosen in an attempt to mimic the clinical scheduling of antibiotics (that is, cell therapy is just like the drug to be used in our daily clinical practice) for patients with sepsis syndrome.

### Isolation of adipose-derived mesenchymal stem cells from Sprague–Dawley rats

Rats in groups 4 and 5 were anesthetized with inhalational 2% isoflurane 14 days before induction of acute kidney IR injury for harvest of peri-epididymal adipose tissue, as we reported previously [5,6]. In detail, the adipose tissue surrounding the epididymis was carefully dissected and excised. Then 200 to 300 μl sterile saline was added to every 0.5 g tissue to prevent dehydration. The tissue was cut into <1 mm^3^ size pieces using sharp, sterile surgical scissors. Sterile saline (37°C) was added to the homogenized adipose tissue in a ratio of 3:1 (saline:adipose tissue), followed by the addition of stock collagenase solution to a final concentration of 0.5 units/ml. The tubes with the contents were placed and secured on a Thermaline shaker and incubated with constant agitation for 60 ± 15 minutes at 37°C. After 40 minutes of incubation, the content was triturated with a 25 ml pipette for 2 to 3 minutes. The cells obtained were placed back on the rocker for incubation. The contents of the flask were transferred to 50 ml tubes after digestion, followed by centrifugation at 600×*g* for 5 minutes at room temperature. The fat layer and saline supernatant from the tube were poured out gently in one smooth motion or were removed using vacuum suction. The cell pellet thus obtained was resuspended in 40 ml saline and then centrifuged again at 600×*g* for 5 minutes at room temperature. After being resuspended again in 5 ml saline, the cell suspension was filtered through a 100 μm filter into a 50 ml conical tube to which 2 ml saline was added to rinse the remaining cells through the filter. The flow-through was pipetted to a 40 μm filter into a new 50 ml conical tube. The tubes were centrifuged for a third time at 600×*g* for 5 minutes at room temperature. The cells were resuspended in saline.

**Figure 1:**
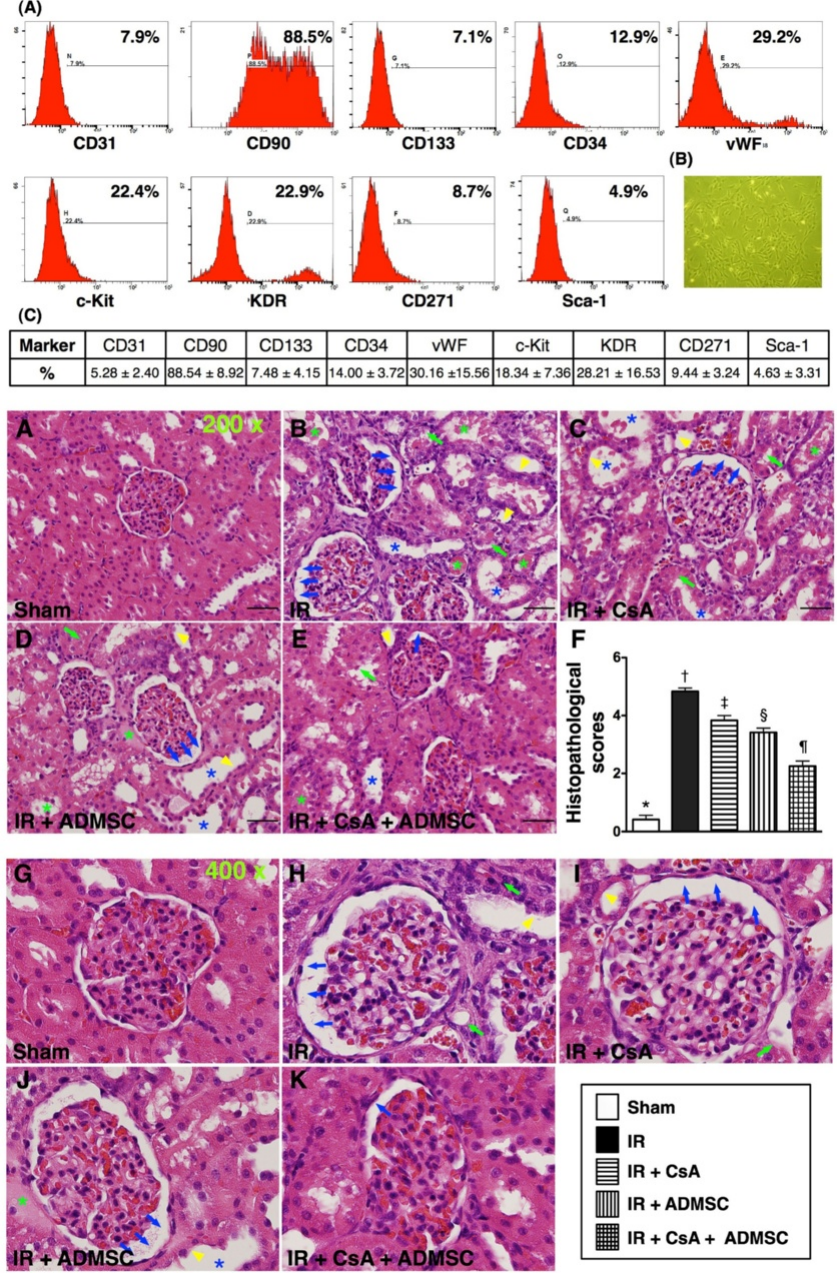
**Flow cytometry and microscopy analysis of rat adipose-derived mesenchymal stem cells and histopathological scoring of renal injury.** Flow cytometric analysis and microscopy findings of rat adipose-derived mesenchymal stem cells (ADMSCs) (*n* = 6) and histopathological scoring of acute kidney ischemia–reperfusion (IR) injury at 72 hours after the IR procedure (*n* = 8). Upper panel: (**A**) Flow cytometric results of rat ADMSCs and endothelial progenitor cells (EPC) on day 14 after cell culturing showed that CD90^+^ cells contributed the highest population of stem cells (*n* = 6). (**B**) Spindle-shaped morphology of stem cells illustrated in the lower right corner (200×). (**C**) Percentage of ADMSCs and EPC shown was the mean value of *n* = 6. Lower panel: (**A**) to (**D**), (**G**) to (**K**) H & E staining (200× in (**A**) to (**E**), and 400× in (**G**) to (**K**)) of kidney sections in sham control, IR, IR + cyclosporine (CsA), IR + ADMSCs and IR + CsA + ADMSCs, showing significantly higher degree of loss of brush border in renal tubules (yellow arrowheads), cast formation (green asterisk), tubular dilatation (blue asterisk), tubular necrosis (green arrows), and dilatation of Bowman’s capsule (blue arrows) in the IR without treatment group than in other groups at 72 hours after IR procedure. (**F**) *P <0.0001 vs. other groups with different symbols; † vs. ‡ vs. ¶ vs. §, all *P* <0.05 for inter-group comparisons. All statistical analyses were with one-way analysis of variance followed by Bonferroni multiple comparison *post-hoc* test. Different symbols (*, †, ‡, ¶, §) among different groups indicate significance (at 0.05 level). Scale bars in right lower corner = 50 μm.

Isolated ADMSCs were cultured in a 100 mm diameter dish with 10 ml DMEM culture medium containing 10% fetal bovine serum for 14 days (see Figure 1B for microscopy). Flow cytometric analysis was performed to identify cellular characteristics after cell-labeling with appropriate antibodies on day 14 of cell cultivation 30 minutes prior to implantation (Figure 1A,B,C).

### ADMSC labeling and acute kidney ischemia–reperfusion injury protocol

On day 14, CM-Dil (Vybrant™ Dil cell-labeling solution, 50 μg/ml; Molecular Probes, Inc., Carlsbad, CA, USA) was added to the culture medium 30 minutes before the acute kidney IR procedure for ADMSC labeling as reported previously [5]. After ADMSC labeling, all animals were anesthetized by inhalational 2.0% isoflurane, and placed supine on a warming pad at 37°C for midline laparotomies; left nephrectomies were performed in groups 2, 3, 4 and 5 but sham-operated rats (group 1) received laparotomy alone. Acute right kidney IR injury was then conducted in all animals except sham controls by clamping the right renal pedicle for 1 hour using nontraumatic vascular clips before reperfusion for 72 hours.

Group 2 animals received an intravenous injection of 500 μl DMEM medium only 1 hour after the ischemia procedure, followed by intravenous injections of 500 μl DMEM medium at 6 and 24 hours after the IR procedure through the penile vein. Group 4 and 5 animals underwent the same protocol, except an equal volume of DMEM medium (500 ml) with syngeneic ADMSCs (1.2 × 10^6^) was administered at each time point instead of pure culture medium. All animals were sacrificed 72 hours after the IR procedure.

### Measurement of renal function before and after ischemia–reperfusion procedure

Blood samples that were collected before and after (at 24 and 72 hours) the IR procedure before their sacrifice were then stored at −80°C until analyses of serum blood urea nitrogen (BUN), urine protein and urine creatinine were performed at the end of the experiment for all animals. The concentrations of these biomarkers were assessed in duplicate with a commercially available assay kit (BioAssay Systems, Hayward, CA, USA). Intra-individual variability in creatinine and BUN level were assessed in each group. The mean intra-assay coefficients of variance for creatinine and BUN were all <4.0%, respectively.

### Collection of 24-hour urine before and after (on days 1 and 3) ischemia–reperfusion procedure

Twenty-four-hour urine was collected in all animals prior to the IR procedure and at 24 and 72 hours after the IR procedure before their sacrifice to determine the daily urine volume and to measure the ratio of urine protein to urine creatinine excretion.

### Histopathology scoring at 72 hours after the ischemia–reperfusion procedure

Histopathology scoring was determined in blinded fashion as reported previously [5,31]. Briefly, the right kidney specimens from all animals were fixed in 10% buffered formalin, embedded in paraffin, sectioned at 5 μm and stained (H & E) for light microscopy. The score reflected the grading of tubular necrosis, loss of brush border, cast formation and tubular dilatation in 10 randomly chosen, nonoverlapping fields (200×) as follows: 0, none; 1, ≤10%; 2, 11 to 25%; 3, 26 to 45%; 4, 46 to 75%; and 5, ≥76%.

### Immunofluorescent and immunohistochemical studies

The immunofluorescent (IF) and immunohistochemical (IHC) methodologies used in this study have recently been described in detail [5]. Briefly, CM-Dil-positive ADMSCs, which were engrafted in the right renal parenchyma, were determined using the IF staining methodology that we also used for the examination of heme oxygenase (HO)-1-positive cells and CD68^+^ cells (an indicator of macrophage) using respective primary antibodies. Moreover, our IHC labeling technique was adopted to identify glutathione peroxidase (GPx)-positive, glutathione reductase (GR)-positive and proliferating cell nuclear antigen-positive cells using respective primary antibodies based on our recent study [32]. Irrelevant antibodies were used as controls in the current study. Additionally, an IHC-based scoring system was conducted in blinded fashion for semiquantitative analyses of GR and GPx as a percentage of positive cells. Scoring of positively-stained cells for GR and GPx was as follows: 0, no stain; 1, <15%; 2, 15 to 25%; 3, 25 to 50%; 4, 50 to 75%; 5, >75 to 100% per high-power field; 200×).

### Western blot analysis of right kidney specimens

Our methods were previously detailed in our recent reports [5-7,21]. mAbs against intercellular adhesion molecule-1 (1:2000; Abcam, Cambridge, MA, USA), NAD(P)H quinone oxidoreductase (NQO)-1 (1:1,000; Abcam, Cambridge, MA, USA), HO-1 (1:250; Abcam), and polyclonal antibodies against TNFα (1:1,000; Cell Signaling, Danvers, MA, USA), NF-κB (1:250; Abcam, Cambridge, MA, USA), platelet-derived growth factor (1:500; Abcam, Cambridge, MA, USA), NADPH oxidase (NOX)-1 (1:1,500; Sigma, St. Louis, MO, USA), NOX-2 (1:500; Sigma, St. Louis, MO, USA), Bax (1:1,000; Abcam, Cambridge, MA, USA), caspase 3 (1:1,000; Cell Signaling, Danvers, MA, USA), poly(ADP-ribose) polymerase (PARP) (1:1,000; Cell Signaling, Danvers, MA, USA), Bcl-2 (1:250; Abcam, Cambridge, MA, USA), cytosolic cytochrome C (1:2,000; BD, San Jose, CA, USA), mitochondrial cytochrome C (1:2,000; BD) and endothelial nitric oxide synthase (eNOS) (1:1,000; Abcam, Cambridge, MA, USA) were used. Signals were detected with horseradish peroxidase-conjugated goat anti-mouse, goat anti-rat, or goat anti-rabbit IgG.

The Oxyblot Oxidized Protein Detection Kit was purchased from Chemicon (S7150, Billerica, MA, USA). The procedure was the same as we reported previously [5,6,21]. A standard control was loaded on each gel.

### Real-time quantitative PCR analysis

The mRNA expressions of caspase 3, Bcl-2, matrix metalloproteinase-9, TNFα, NF-κB, RANTES, NOX-1, NOX-2, HO-1, NQO-1, IL-10, prostaglandin E2 and eNOS in each of the five groups of animals were analyzed with quantitative real-time PCR and compared. Technical details were according to our previous reports [5-7,21].

### Superoxide dismutase assay

After weighing, the right kidney was sliced, homogenized in lysis buffer (100 μM Tris–HCl, pH 7.4 containing 0.5% Triton, 5 mM β-mercaptoethanol, 0.1 mg/ml phenylmethylsulfonyl fluoride) with the Dounce homogenizer and then centrifuged at 14,000×*g* for 5 minutes to remove tissue debris. The supernatant was collected and the protein concentration determined with the BCA protein assay kit (Pierce, Rockford, IL, USA). The final total protein concentration was adjusted to 20 mg/ml. To measure superoxide dismutase (SOD) activity, 20 μl (containing 400 μg protein) was applied for enzymatic reaction with a commercial SOD assay kit (#K335-100; BioVision, Mountain View, CA, USA). Sample preparation and calculation of SOD activity were performed according to the manufacturer’s instructions.

### Statistical analysis

Quantitative data are expressed as the mean ± standard deviation. Statistical analyses were performed using SAS statistical software for Windows version 8.2 (SAS Institute, Cary, NC, USA) to conduct analysis of variance followed by Bonferroni multiple-comparison *post-hoc* test. *P* <0.05 was considered statistically significant.

## Results

### Time courses of circulating levels of creatinine and blood urea nitrogen, and the ratio of urine protein to creatinine after acute kidney IR procedure

Three time points (before, 24 hours after and 72 hours after the acute kidney IR procedure) were chosen to evaluate serial changes in serum levels of creatinine and BUN (Table 1). Creatinine and BUN levels did not differ between the five groups prior to the IR procedure. However, both BUN and creatinine levels were significantly higher in IR (group 2) than in normal controls (group 1), IR + CsA (group 3), IR + ADMSC (group 4) and IR + CsA-ADMSC (group 5), significantly higher in groups 3, 4 and 5 than in group 1, but not different amongst groups 3, 4 and 5 at 24 hours after the IR procedure (Table 1). By 72 hours after the IR procedure, serum creatinine was highest in group 2 and lowest in group 1, significantly higher in groups 3 and 4 than in group 5, but not different between groups 3 and 4. The BUN level by 72 hours was lowest in group 1 and highest in group 2, significantly higher in group 3 than in groups 4 and 5, but similar in groups 4 and 5 (Table 1). These findings implicated that either CsA or ADMSC therapy significantly protected, and combined therapy with CsA-ADMSC more significantly protected, renal function after IR injury.

**Table 1:** Time courses for circulating levels of creatinine and BUN, and the urine protein:creatinine ratio

**Variable**	**Group 1**	**Group 2**	**Group 3**	**Group 4**	**Group 5**	***P *** **value**
**At 0 hours**						
Serum creatinine (mg/dl)	0.21 ± 0.05	0.22 ± 0.03	0.19 ± 0.03	0.23 ± 0.04	0.19 ± 0.05	0.244
Serum BUN (mg/dl)	14.8 ± 2.1	16.3 ± 3.3	13.5 ± 1.1	17.1 ± 1.7	16.4 ± 2.2	0.062
Ratio of urine protein to creatinine	1.10 ± 0.17	1.14 ± 0.53	1.14 ± 0.32	1.35 ± 0.90	1.18 ± 0.34	0.924
**At 24 hours**						
Serum creatinine (mg/dl)	0.27 ± 0.08*	3.28 ± 1.29^†^	1.72 ± 1.93^‡^	1.55 ± 1.61^‡^	1.6 ± 0.72^‡^	<0.001
Serum BUN (mg/dl)	16.6 ± 3.3*	86.7 ± 15.9^†^	67.8 ± 37.4^‡^	68.0 ± 35.9^‡^	68.0 ± 35.9^‡^	<0.001
Ratio of urine protein to creatinine	1.28 ± 0.49*	6.75 ± 2.92^†^	4.01 ± 0.80^‡^	3.27 ± 0.33^§^	2.73 ± 0.60^¶^	<0.001
**At 72 hours**						
Serum creatinine (mg/dl)	0.44 ± 0.06*	3.58 ± 3.16^†^	1.21 ± 0.83^‡^	1.27 ± 0.83^‡^	0.82 ± 0.32^§^	<0.001
Serum BUN (mg/dl)	23.3 ± 2.2*	109 ± 89.7^†^	63.5 ± 37.4^‡^	49.9 ± 29.5^§^	47.7 ± 18.3^§^	<0.001
Ration of urine protein to creatinine	1.14 ± 0.60*	3.70 ± 2.82^†^	2.08 ± 2.06^‡^	1.52 ± 0.34^§^	1.19 ± 0.23^¶^	<0.001

Time courses of circulating levels of creatinine and blood urea nitrogen (BUN), and the ratio of urine protein to creatinine after acute kidney ischemia–reperfusion procedure (*n* = 8 in each group). Data are expressed as mean ± standard deviation. Group 1 = sham control; group 2 = ischemia reperfusion (IR); group 3 = IR + cyclosporine (CsA); group 4 = IR + adipose-derived mesenchymal stem cells (ADMSC); group 5 = IR + CsA + ADMSC. Statistical analysis in each group by analysis of variance followed by Bonferroni multiple comparison *post-hoc* test. Symbols (*, †, ‡, §, ¶) indicate significance (at 0.05 level) by Bonferroni multiple-comparison *post-hoc* test: † vs. ‡ vs. § vs. ¶, all *P* <0.05 for inter-group comparisons.

The ratio of urine protein to urine creatinine was similar in the five groups prior to the IR procedure. However, at 24 and 72 hours after IR, this parameter was highest in group 2 and lowest in group 1, significantly higher in group 3 than in groups 4 and 5, and significantly higher in group 4 than group 5 (Table 1).

### Changes in mRNA expressions of apoptotic, inflammatory, oxidative, antioxidant, and anti-inflammatory mediators in renal parenchyma

From tissues harvested at 72 hours post IR injury, the mRNA expression of caspase 3, an index of apoptosis, was highest in group 2 and lowest in group 1, significantly higher in groups 3 and 4 than in group 5, but similar in groups 3 and 4. Conversely, the pattern of mRNA expression of Bcl-2, an index of anti-apoptosis, was opposite to that of Bax mRNA expression in the five groups (Table 2).

**Table 2:** mRNA expression of apoptotic, inflammatory, oxidative, antioxidant, and anti-inflammatory mediators in renal parenchyma after injury

**Variable**	**Group 1**	**Group 2**	**Group 3**	**Group 4**	**Group 5**	***P *** **value**
Caspase 3	1*	1.24 ± 0.15^†^	0.91 ± 0.09^‡^	0.88 ± 0.07^‡^	0.6 ± 0.06^§^	<0.0001
Bcl-2	1*	0.60 ± 0.06^†^	0.73 ± 0.06^‡^	0.77 ± 0.06^‡^	0.94 ± 0.09^§^	<0.0009
TNF-α	1*	1.21 ± 0.09^†^	0.88 ± 0.07^‡^	0.84 ± 0.04^‡^	0.56 ± 0.07^§^	<0.0001
MMP-9	1*	1.32 ± 0.1^†^	0.94 ± 0.08^‡^	0.92 ± 0.04^‡^	0.69 ±0.05^§^	<0.0001
RANTES	1*	1.30 ± 0.04^†^	1.03 ± 0.04^‡^	1.08 ± 0.06^‡^	0.84 ± 0.07^§^	<0.003
NOX-1	1*	1.30 ± 0.09^†^	1.07 ± 0.05^‡^	1.03 ± 0.04^‡^	0.89 ± 0.05^§^	<0.007
HO-1	1*	1.43 ± 0.10^†^	1.66 ± 0.09^‡^	1.92 ± 0.11^§^	2.32 ± 0.11^¶^	<0.0001
NQO 1	1*	1.1 ± 0.07^†^	1.22 ± 0.09^‡^	1.32 ± 0.04^§^	1.44 ± 0.10^¶^	<0.0001
IL-10	1*	1.66 ± 0.07^†^	1.60 ± 0.11^‡^	2.09 ± 0.06^§^	2.75 ± 0.11^¶^	<0.0001
PGE-2	1*	0.82 ± 0.10^†^	1.05 ± 0.07^‡^	1.17 ± 0.05^§^	1.29 ± 0.07^¶^	<0.0001
eNOS	1*	0.62 ± 0.04^†^	0.75 ± 0.05^‡^	0.89 ± 0.05^§^	1.11 ± 0.04*	<0.0001

Relative changes in mRNA expression of apoptotic, inflammatory, oxidative, antioxidant, and anti-inflammatory mediators in renal parenchyma after ischemia–reperfusion injury (*n* = 8 in each group). Data expressed as mean ± standard deviation. eNOS, endothelial nitric oxide synthase; HO-1, heme oxygenase 1; MMP, matrix metalloproteinase; NOX, NAD(P)H oxidase; NQO, NAD(P)H quinone oxidoreductase; PGE2, prostaglandin E2; RANTES, regulated and normal T-cell expressed and presumably secreted. Group 1 = sham control; group 2 = ischemia–reperfusion (IR); group 3 = IR + cyclosporine (CsA); group 4 = IR + adipose-derived mesenchymal stem cells (ADMSC); group 5 = IR + CsA + ADMSC. Statistical analysis in each group by analysis of variance followed by Bonferroni multiple comparison *post-hoc* test. Symbols (*, †, ‡, §, ¶) indicate significance (at 0.05 level) by Bonferroni multiple-comparison *post-hoc* test: † vs. ‡ vs. § vs. ¶, all *P* <0.05 for inter-group comparisons.

From tissues harvested at 72 hours post IR injury, the mRNA expression of TNFα, matrix metalloproteinase-9 and RANTES, three indicators of inflammation, were highest in group 2 and lowest in group 1, significantly higher in groups 3 and 4 than in group 5, but not significantly different between groups 3 and 4; this pattern was identical for the mRNA expressions of NOX-1 and NOX-2, two oxidative stress biomarkers. Conversely, the patterns of mRNA expressions of HO-1 and NQO 1, two antioxidant/antioxidative stress biomarkers, were opposite to those of NOX-1 and NOX-2 in the five groups (Table 2).

From tissues harvested at 72 hours post IR injury, the mRNA expression of IL-10, an anti-inflammatory biomarker, was lowest in group 1 and highest in group 5, significantly higher in groups 3 and 4 than in group 2, and significantly higher in group 4 than in group 3. Additionally, the mRNA expression of prostaglandin E2, an anti-inflammatory mediator, was lowest in group 2 and highest in group 5, significantly higher in group 4 than in groups 1 and 3, but similar in groups 1 and 3. Furthermore, the mRNA expression of eNOS, an index of anti-inflammatory mediator/endothelial cell integrity, was lowest in group 2 and highest in groups 1 and 5, significantly higher in group 4 than in group 3, but similar in groups 1 and 5 (Table 2).

### Flow cytometric analysis and histopathology of kidney

The upper panel of Figure 1 (Figure 1A,B,C) illustrated the flow cytometric results of rat ADMSCs and endothelial progenitor cells (EPCs) on day 14 after cell culturing. The results showed that CD90^+^ cells contributed the highest population of stem cells.

To determine the effects of CsA, ADMSC transplantation and a combination of these treatments on IR-induced renal injury, a histological scoring system based on the typical microscopic features of acute tubular damage (including extensive tubular necrosis and dilatation, cast formation and loss of brush border) was adopted (Figure 1, lower panel). At 72 hours after the IR procedure, this injury score was highest in group 2, significantly higher in group 3 than in groups 1, 4 and 5, significantly higher in group 4 than in groups 1 and 5, and significantly higher in group 5 than in group 1. These findings suggest that CsA monotherapy significantly protected the kidney from IR damage, but that ADMSC therapy offered significantly more protection than CsA. Combination therapy using CsA plus ADMSCs, however, offered significantly better protection than either of these monotherapies.

### Infiltrated CD68^+^ cells and superoxide dismutase activity in kidney

**Figure 2:**
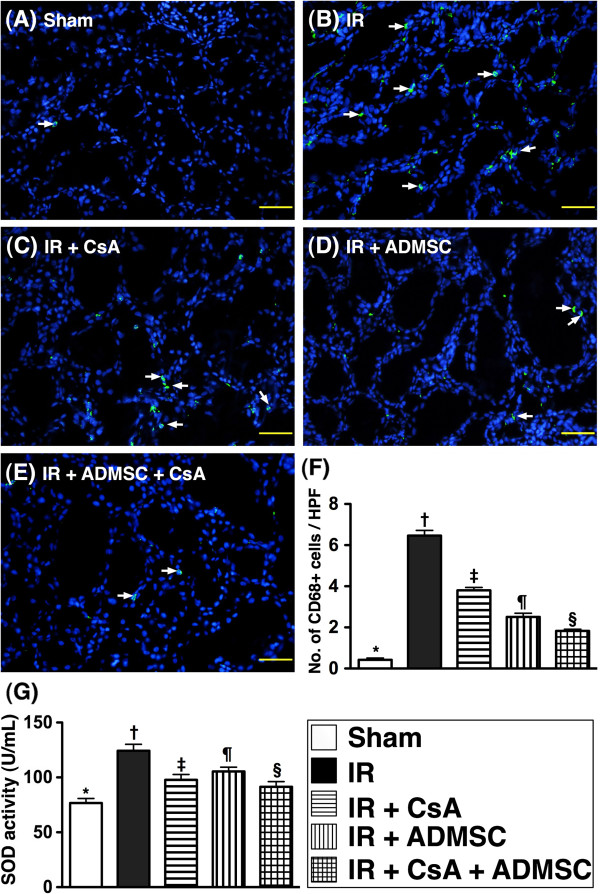
**Immunofluorescent staining for inflammatory cells and enzymatic analysis of superoxide dismutase activity.** Immunofluorescent (IF) staining for inflammatory cells and enzymatic analysis of superoxide dismutase (SOD) activity at 72 hours after the ischemia–reperfusion (IR) procedure (*n* = 8). (**A**) to (**E**) High-power field (HPF) IF microscopic findings (200×) showing the number of CD68^+^ cell infiltration (white arrows) in the five groups. Scale bars in right lower corner = 50 μm. (**F**) *P <0.0001 vs. other groups with different symbols; † vs. ‡ vs. ¶ vs. §, all *P* <0.05 for inter-group comparisons. (**G**) Analytical result of SOD activity in renal parenchyma at 72 hours after the IR procedure. *P <0.0001 vs. other groups with different symbols; † vs. ‡ vs. ¶ vs. §, all *P* <0.05 for inter-group comparisons. All statistical analyses were with one-way analysis of variance followed by Bonferroni multiple comparison *post-hoc* test. Different symbols among different groups indicate significance (at 0.05 level). ADMSC, adipose-derived mesenchymal stem cell; CsA, cyclosporine A.

From tissues harvested at 72 hours post IR injury, the number of CD68^+^ cells (that is, macrophages), an index of inflammation, was highest in group 2 and lowest in group 1, significantly higher in groups 3 and 4 than in group 5, and significantly higher in group 3 than in group 4. Additionally, levels of SOD activity, an indicator of oxidative stress, showed a similar pattern except for a significant reverse relationship between groups 3 and 4 (Figure 2).

### Expressions of antioxidant activity and vascular density in kidney

**Figure 3:**
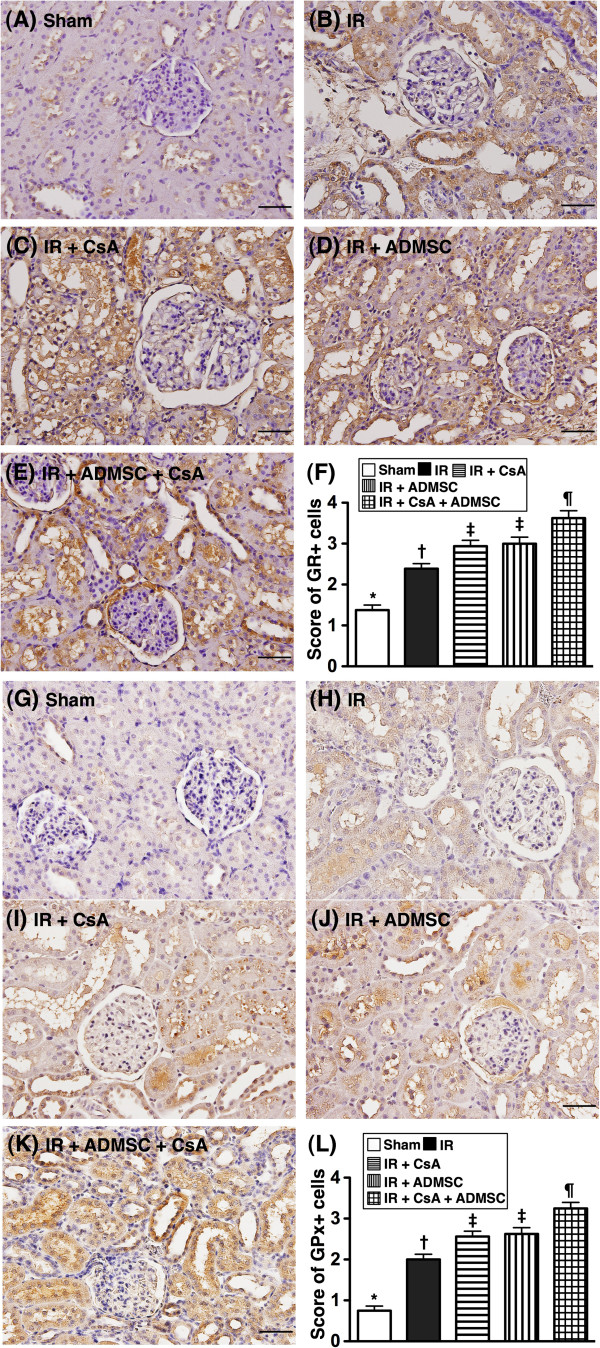
**Immunohistochemical staining for renal expressions of antioxidative markers at 72 hours after ischemia–reperfusion procedure.** (**A**) to (**E**) Microscopic findings of immunohistochemical (IHC) stain (*n* =8, 200×) for glutathione reductase (GR)-positive cells (brown) in renal parenchyma of five groups. Scale bars in right lower corner = 50 μm. (**F**) *P <0.001 vs. other groups with different symbols; † vs. ‡ vs. ¶, all *P* <0.05 for inter-group comparisons. (**G**) to (**K**) Microscopic findings of IHC stain (200×) for glutathione peroxidase (GPx)-positive cells (brown) renal parenchyma of five groups. Scale bars in right lower corner = 50 μm. (**L**) *P <0.001 vs. other groups with different symbols; † vs. ‡ vs. ¶, all *P* <0.05 for inter-group comparisons. All statistical analyses performed using one-way analysis of variance followed by Bonferroni multiple comparison *post-hoc* test. Different symbols (*, †, ‡, ¶) among different groups indicate significance (at 0.05 level). ADMSC, adipose-derived mesenchymal stem cell; CsA, cyclosporine A.

**Figure 4:**
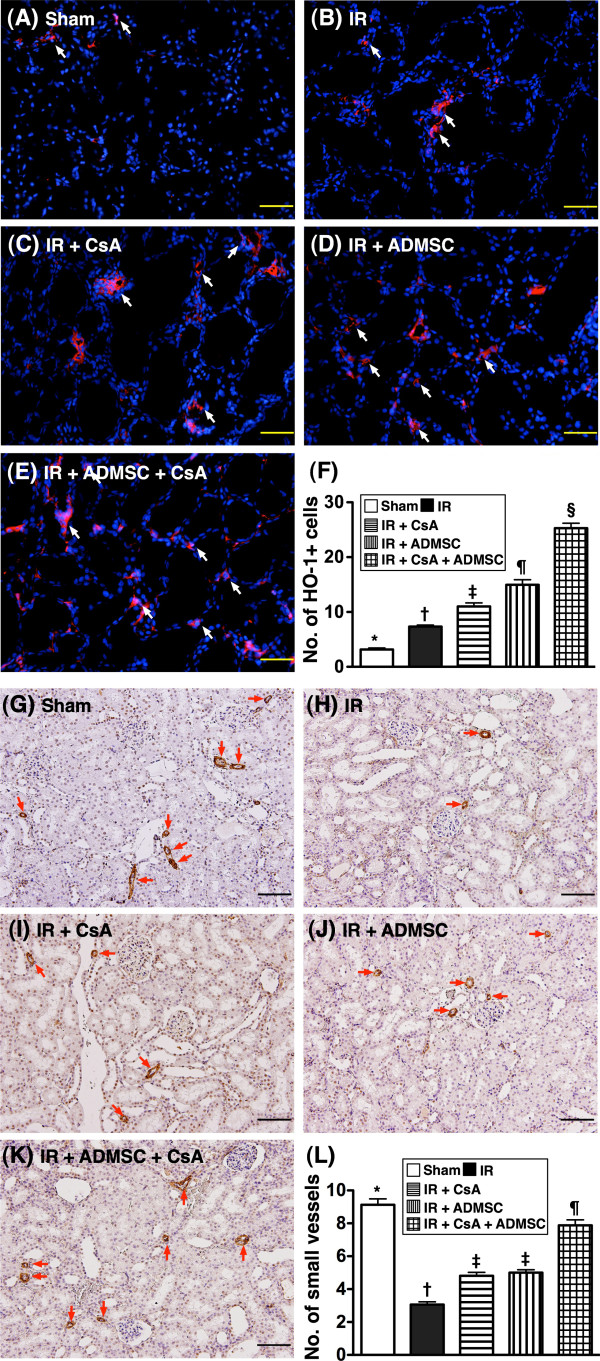
**Immunofluorescent staining for renal heme oxygenase-1 antioxidant and alpha smooth muscle actin expressions.** Immunofluorescent (IF) staining for renal heme oxygenase (HO)-1 antioxidant and alpha smooth muscle actin (α-SMA) expressions at 72 hours after ischemia–reperfusion (IR) (*n* = 8). (**A**) to (**E**) Microscopic findings of IF staining (200×) for number of HO-1^+^ cells (white arrows) in renal parenchyma of five groups. Scale bars in right lower corner = 50 μm. (**F**) *P <0.0001 vs. other groups with different symbols; † vs. ‡ vs. ¶ vs. §, all *P* <0.05 for inter-group comparisons. (**G**) to (**K**) Microscopic (100×) findings of α-SMA staining for the number of small vessels (defined as diameter <25 μm; red arrows) among five groups. (**L**) *P <0.001 vs. other groups with different symbols; † vs. ‡ vs. ¶, all *P* <0.05 for inter-group comparisons. Scale bars at right lower corner = 100 μm (100×). All statistical analyses performed using one-way analysis of variance followed by Bonferroni multiple comparison *post-hoc* test. Different symbols (*, †, ‡, ¶, §) among different groups indicate significance at the 0.05 level. ADMSC, adipose-derived mesenchymal stem cell; CsA, cyclosporine A.

From tissues harvested at 72 hours post IR injury, IHC staining demonstrated that the expressions of GR (Figure 3A to F) and GPx (Figure 3G to L), two oxidoreductase enzymes, were highest in group 5 and lowest in group 1, significantly higher in groups 3 and 4 than in group 2, but similar in groups 3 and 4. Additionally, IF staining revealed that the number of HO-1-positive cells, another indicator of oxidoreductase enzyme, exhibited an identical pattern to GR and GPx IHC staining in the five groups (Figure 4A to F).

Staining for α-smooth muscle actin showed that the number of small vessels in kidney parenchyma was highest in group 1 and lowest in group 2, significantly higher in group 5 than in groups 3 and 4, but similar in groups 3 and 4 (Figure 4G to L).

### Angiogenesis biomarkers of kidney

**Figure 5:**
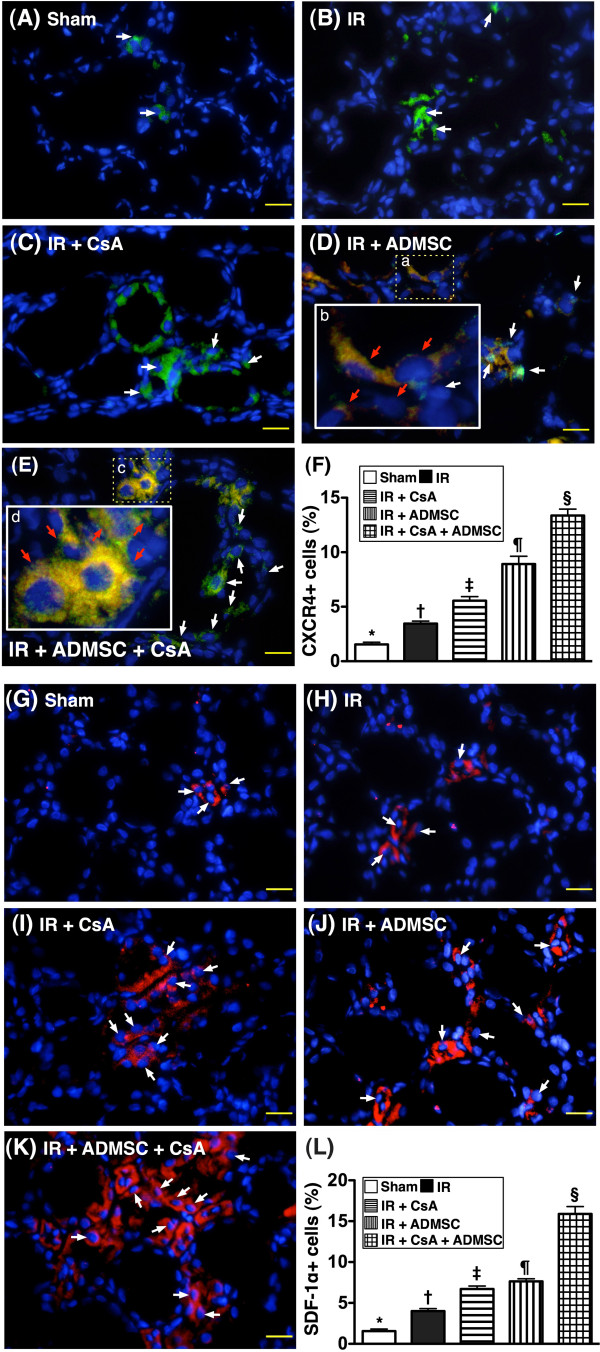
**Engraftment of adipose-derived mesenchymal stem cells in renal tissue at 72 hours after ischemia–reperfusion.** (**A)** to (**E**) Identification of double-stained adipose-derived mesenchymal stem cells (ADMSCs) (Dil dye + CXCR4^+^ cells; red arrows) and single stain for CXCR4^+^ cells (white arrows) (400×) in peri-tubular and interstitial areas of kidney 72 hours post ischemia–reperfusion (IR) procedure among five groups (*n* = 8). (**D**), (**E**) Merged image from double-staining (Dil + CXCR4) showing cellular elements of mixed green and yellow under higher magnifications ((**B**) and (**D**) are magnified images of (**A**) and (**C**), respectively), indicating implanted CXCR4^+^ cells in lung parenchyma. DAPI counter-staining for nuclei (blue). Scale bars at right lower corner = 20 μm. (**F**) *P <0.0001 vs. other groups with different symbols; † vs. ‡ vs. ¶ vs. §, all *P* <0.05 for inter-group comparisons. (**G**) to (**K**) Identification of single stain for stromal cell-derived factor (SDF)-1α^+^ cells (red color; white arrows) (400×) in peri-tubular area and interstitial area of kidney 72 hours post IR procedure among five groups. DAPI counter-staining for nuclei (blue). Scale bars at right lower corner = 20 μm. (**L**) *P <0.0001 vs. other groups with different symbols; † vs. ‡ vs. ¶ vs. §, all *P* <0.05 for inter-group comparisons. All statistical analyses performed using one-way analysis of variance followed by Bonferroni multiple comparison *post-hoc* test. Different symbols (*, †, ‡, ¶, §) among different groups indicate significance at the 0.05 level. CsA, cyclosporine A.

**Figure 6:**
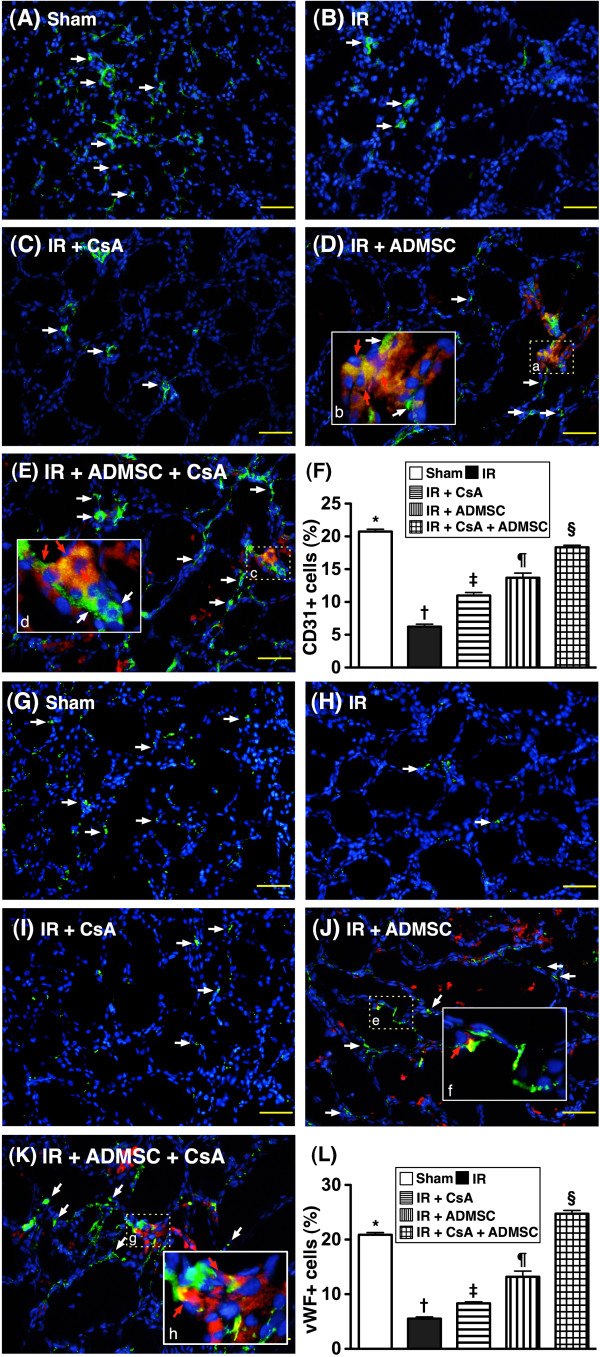
**Immunofluorescent stains for presence of CD31**^**+ **^**and vWF**^**+ **^**cells in renal parenchyma after ischemia–reperfusion.** Immunofluorescent (IF) stains for presence of CD31^+^ and von Willebrand Factor (vWF)^+^ cells in renal parenchyma at 72 hours after ischemia–reperfusion (IR) (*n* = 8). (**A**) to (**E**) IF staining (200×) of CD31^+^ cells (white) in renal parenchyma. Notably fewer CD31^+^ cells in the IR group (**B**) than in other groups (**A**, **C**, **D**, **E**). (**D**), (**E**) Merged image from double-staining (DiI + CD31) showing cellular elements of mixed red and yellow (red arrows) under higher magnifications ((**B**) and (**D**) are magnified images of (**A**) and (**C**), respectively), indicating implanted numerous doubly-stained cells (CD31^+^) in peri-tubular and interstitial areas. DAPI counter-staining for nuclei (blue). Scale bars at right lower corner = 50 μm. **F**) *P <0.0001 vs. other groups with different symbols; † vs. ‡ vs. ¶ vs. §, all *P* <0.05 for inter-group comparisons. (**G**) to (**K**) IF staining (200×) of vWF^+^ cells (white) in renal parenchyma. Notably fewer vWF^+^ cells in the IR group (**H**) than in other groups (**G**, **I**, **J**, **K**). (**K**) Merged image from double-staining (DiI + vWF) showing cellular elements of mixed red and yellow (red arrows) under higher magnifications ((**F**) and (**H**) are magnified images of (**E**) and (**G**), respectively), indicating implanted doubly-stained cells (vWF^+^) in peri-tubular and interstitial areas. DAPI counter-staining for nuclei (blue). Scale bars at right lower corner = 50 μm. (**L**) *P <0.0001 vs. other groups with different symbols; † vs. ‡ vs. ¶ vs. §, all *P* <0.05 for inter-group comparisons. All statistical analyses performed using one-way analysis of variance followed by Bonferroni multiple comparison *post-hoc* test. Different symbols (*, †, ‡, ¶, §) among different groups indicate significance at the 0.05 level. ADMSC, adipose-derived mesenchymal stem cells; CsA, cyclosporine A.

From tissues harvested at 72 hours post IR injury, the number of CXCR4^+^ cells (Figure 5A to F) and SDF-1α^+^ cells (Figure 5G to L), two indices of EPC surface markers, were highest in group 5, lowest in group 1, significantly lower in group 2 than in groups 3 and 4, and significantly lower in group 3 than in group 4. Additionally, CD31^+^ cells (Figure 6A to F) and vWF^+^ cells (Figure 6G to L), two indicators of endothelial cell surface markers, were highest in group 5 and lowest in group 2, significantly higher in group 1 than in groups 3 and 4, and significantly higher in group 4 than in group 3.

### Molecular-cellular damaged biomarkers in kidney

**Figure 7:**
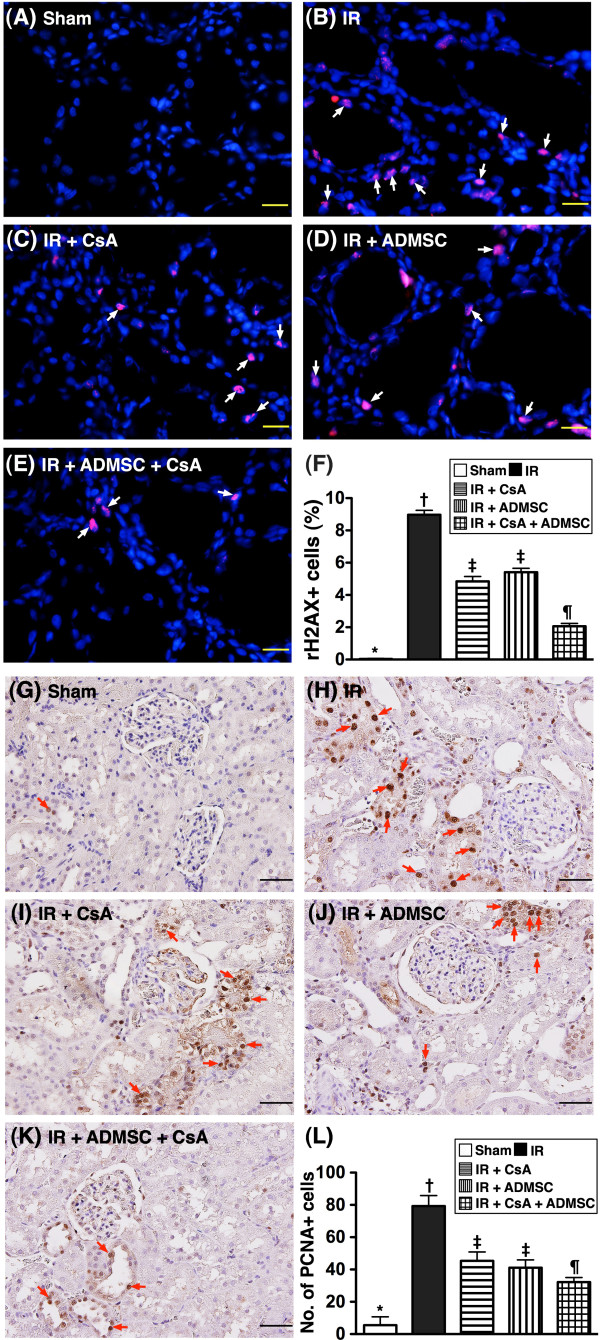
**Immunofluorescent and immunohistochemical stains of DNA damage cellular markers in renal parenchyma after ischemia–reperfusion.** Immunofluorescent (IF) and immunohistochemical (IHC) stains of DNA damage cellular markers in renal parenchyma at 72 hours after ischemia–reperfusion (IR) *(n* = 8). (**A**) to (**E**) Microscopic findings of IF stain (400×) for γH2AX^+^ cells (white arrows) in renal parenchyma of five groups. DAPI counter-staining for nuclei (blue). Scale bars in right lower corner = 20 μm. (**F**) *P <0.0001 vs. other groups with different symbols; † vs. ‡ vs. ¶, all *P* <0.05 for inter-group comparisons. (**G**) to (**K**) Microscopic findings of IF stain (200×) for proliferating cell nuclear antigen-positive (PCNA^+^) cells (red arrows) in renal parenchyma of five groups. DAPI counter-staining for nuclei (blue). Scale bars in right lower corner = 50 μm. (**L**) *P <0.0001 vs. other groups with different symbols; † vs. ‡ vs. ¶, all *P* <0.05 for inter-group comparisons. All statistical analyses performed using one-way analysis of variance followed by Bonferroni multiple comparison *post-hoc* test. Different symbols (*, †, ‡, ¶) among different groups indicate significance at the 0.05 level. ADMSC, adipose-derived mesenchymal stem cells; CsA, cyclosporine A.

From tissues harvested at 72 hours post IR injury, IF staining showed that expression of γH2AX-positively stained cells, an index of DNA damage, was highest in group 2 and lowest in group 1, significantly higher in groups 3 and 4 than in group 5, but similar in groups 3 and 4 (Figure 7A to F). IHC staining demonstrated that the number of proliferating cell nuclear antigen-positively stained cells, an indicator of damaged DNA and replication, exhibited an identical pattern to that of γH2AX-positively stained cells in the five groups (Figure 7G to L).

### Protein expressions of inflammatory, reactive oxygen species and cytochrome C biomarkers in kidney

**Figure 8:**
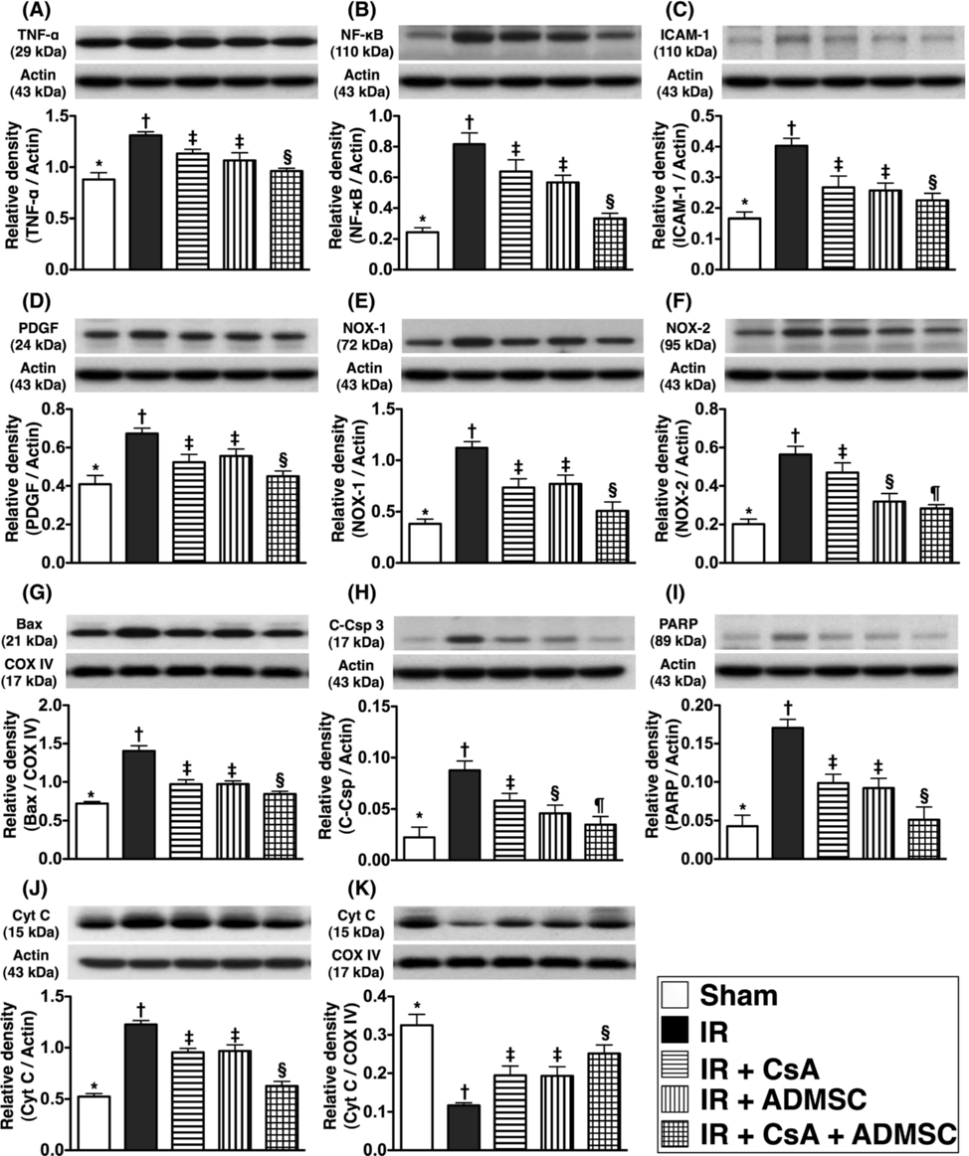
**Protein expression of inflammatory, reactive oxygen species, apoptotic and cytochrome C biomarkers in kidney.** Changes in protein expression of inflammatory, reactive oxygen species (ROS), apoptotic and cytochrome C biomarkers in kidney at 72 hours after ischemia–reperfusion (IR) (*n* = 8). Protein expressions of TNFα (**A**), NF-κB (**B**), intercellular adhesion molecule (ICAM)-1 (**C**) and platelet-derived growth factor (PDGF) (**D**) were highest in the IR group and lowest in the sham control group, significantly higher in IR + cyclosporine A (CsA) and adipose-derived mesenchymal stem cell (ADMSC) groups than in the IR + CsA–ADMSC group. *P <0.001 vs. other groups with different symbols; * vs. † vs. ‡ vs. §, all *P* <0.05 for inter-group comparisons. Protein expressions of NADPH oxidase (NOX)-1 (**E**) and NOX-2 (**F**) were highest in the IR group and lowest in the sham control group, significantly higher in IR + CsA and ADMSC groups than in the IR + CsA–ADMSC group. *P <0.001 vs. other groups with different symbols; † vs. ‡ vs. § vs. ¶, all *P* <0.05 for inter-group comparisons. Protein expressions of mitochondrial Bax (**G**), cleaved caspase 3 (**H**) and cleaved poly(ADP-ribose) polymerase (PARP) (**I**) were highest in the IR group and lowest in the sham control group, significantly higher in IR + CsA and ADMSC groups than in IR + CsA–ADMSC group. For Bax and PARP: *P <0.001 vs. other groups with different symbols; † vs. ‡ vs. §, all *P* <0.05 for inter-group comparisons. The protein expression of cytosolic cytochrome C (**J**) was notably higher whereas mitochondrial cytochrome C (**K**) was markedly lower in the IR group than in other groups. *P <0.001 vs. other groups with different symbols; † vs. ‡ vs. §, all *P* <0.05 for inter-group comparisons. All statistical analyses performed using one-way analysis of variance followed by Bonferroni multiple comparison *post-hoc* test.

To confirm further the expression of inflammatory markers and ROS in IR-injured renal parenchyma, western blot was performed from tissues harvested at 72 hours post IR injury. Results indicated that protein expressions of TNFα, NF-κB, intercellular adhesion molecule-1 and platelet-derived growth factor, four inflammatory biomarkers, were highest in group 2 and lowest in group 1, significantly higher in groups 3 and 4 than in group 5, but similar in groups 3 and 4 (Figure 8A to D). Protein expression of NOX-1, an index of ROS, showed an identical pattern (Figure 8E). Protein expression of NOX-2, another index of ROS, was highest in group 2, lowest in group 1, significantly higher in groups 3 and 4 than in group 5, and significantly higher in group 3 than in group 4 (Figure 8F).

As expected, at 72 hours after the IR procedure, the protein expressions of mitochondrial Bax (Figure 8G) and cleaved PARP (Figure 8I), two indices of apoptotic mediators, were higher in group 2 and lowest in group 1, significantly higher in groups 3 and 4 than in group 5, but similar in groups 3 and 4. Protein expression of cleaved caspase 3 (Figure 8H), another indicator of apoptosis, showed an identical pattern to Bax and PARP in groups 1, 2, 3 and 5; this parameter was also significantly higher in group 3 than in group 4.

From tissues harvested at 72 hours post IR injury, total mitochondrial cytochrome C protein expression was lowest in group 2 and highest in group 1, significantly lower in groups 3 and 4 than in group 5, but similar in groups 3 and 4 (Figure 8K). The total amount of cytosolic cytochrome C protein expression showed a reverse pattern to mitochondrial cytochrome C in the five groups. These findings indicate that the expression of cytochrome C, an index of energy supply and storage in mitochondria, was preserved by either CsA or ADMSC monotherapy but better preserved by combination therapy using CsA and ADMSC (Figure 8J). The highest level of cytosolic cytochrome C in group 2 also suggests significant mitochondrial damage with cytochrome C release into the cytosol in the ischemic kidney.

### Protein expressions of oxidative stress and antioxidant mediators in kidney

**Figure 9:**
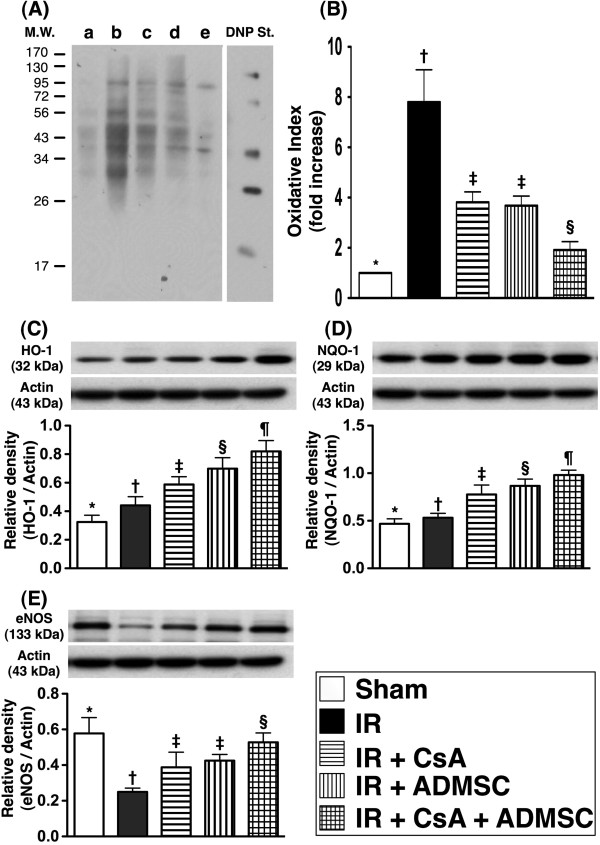
**Western blot results of oxidative stress, antioxidant and endothelial function biomarkers in the kidney.** Western blot results of oxidative stress, antioxidant and endothelial function biomarkers in the kidney at 72 hours after ischemia–reperfusion (IR) (*n* = 8). (**A**) Oxidative index (protein carbonyls) among five groups of animals (a = control, b = IR only, c = IR + CsA, d = IR + ADMSC, e = IR + CsA–ADMSC). (**B**) *P <0.0001 vs. other groups with different symbols; † vs. ‡ vs. §, all *P* <0.05 for inter-group comparisons. (Note: Right lane and left lane shown on the upper panel represent control oxidized molecular protein standard and protein molecular weight marker, respectively.) DNP, 1,3-dinitrophenylhydrazone. (**C**) Protein expressions of heme oxygenase (HO)-1 and (**D**) NAD(P)H quinone oxidoreductase (NQO)-1 among the five groups; *P <0.001 vs. other groups with different symbols; † vs. ‡ vs. § vs. ¶, all *P* <0.05 for inter-group comparisons. (**E**) Protein expression of endothelial nitric oxide synthase (eNOS). *P <0.001 vs. other groups with different symbols; † vs. ‡ vs. §, all *P* <0.05 for inter-group comparisons. Different symbols (*, †, ‡, ¶, §) among different indicate significance at the 0.05 level. ADMSC, adipose-derived mesenchymal stem cells; CsA, cyclosporine A.

From tissues harvested at 72 hours post IR injury, the expression of oxidized protein, an index of oxidative stress, was highest in group 2 and lowest in group 1, significantly higher in groups 3 and 4 than in group 5, but similar in groups 3 and 4 (Figure 9A,B). Additionally, the protein expressions of HO-1 (Figure 9C) and NQO 1 (Figure 9D), two indicators of antioxidants, was lowest in group 1 and highest in group 5, significantly lower in group 2 than in groups 3 and 4, and significantly lower in group 3 than in group 4. The protein expression of eNOS, an indicator of anti-inflammation/integrity of endothelial function, was lowest in group 2 and highest in group 1, significantly higher in group 5 than in groups 3 and 4, and significantly higher in group 4 than in group 3 (Figure 9E).

## Discussion

The results of our study demonstrated that systemic combination therapy using CsA and ADMSC was superior to either monotherapy alone at significantly decreasing IR-induced acute kidney injury by inhibiting IR-triggered macrophage recruitment, DNA damage, inflammation, oxidative stress and ROS generation, and by activating the cellular apoptotic signaling pathway and enhancing angiogenic and antioxidant factors. These findings have important clinical relevance in that CsA and ADMSC combination therapy may quickly serve as a new and promising management strategy against acute kidney injury without ethical barriers, especially in patients rejecting renal transplants.

The most important finding in the present study was that the serum levels of BUN and creatinine, two essential indices of renal function, were substantially increased in acute kidney IR animals compared with sham controls. Additionally, the ratio of urine protein to creatinine, a useful indicator of renal functional impairment, was remarkably increased in acute kidney IR animals compared with sham controls. Furthermore, histopathological renal injury scores were significantly higher in kidney IR animals than in sham controls, but were significantly improved by either CsA or ADMSC monotherapy. Of importance, this study is the first to demonstrate that combination therapy using CsA and ADMSC was superior to either monotherapy alone at reversing impaired renal function and protecting the kidney from acute IR injury.

Previous studies have clearly shown that acute inflammation played an essential role in organ injury in the setting of IR [5,6]. The present study showed that inflammatory mediators (that is, matrix metalloproteinase-9, TNFα, RANTES, NF-κB, intercellular adhesion molecule-1, platelet-derived growth factor, CD68+ cells, SOD activity) were markedly higher in animals that underwent the IR procedure than in animals that did not, consistent with other studies [5,6]. The role of the immunosuppressant/anti-inflammatory CsA has been well investigated [21,23]. Growing data have demonstrated that ADMSCs also have a distinctive capacity to suppress inflammation [5,6,28]. In the present study, the expressions of these inflammatory biomarkers were remarkably reduced equally by either CsA or ADMSC monotherapy, corroborating the findings of others [5,6,21,23,28]. Of particular importance, however, was that combination therapy using CsA and ADMSC provided a significantly more profound effect at reducing inflammatory mediators. This finding lends at least some explanation to why kidney IR injury was attenuated by either monotherapy alone and yet was further attenuated by the co-treatment regimen.

Organ damage from acute IR has been shown principally to result from the ROS burst during reperfusion of ischemic tissues that can trigger the opening of the MPT pore [11-14]. The generation of ROS, in turn, contributes to the production of apoptotic mediators, inflammatory cytokines and oxidative stress [11-14]. One important finding in the current study was that, compared with normal controls, the protein expressions of oxidative stress (that is, oxidized protein), ROS (NOX-1, NOX-2) and enzymatic analysis of SOD activity were significantly enhanced in animals after acute kidney IR without treatment. Additionally, the apoptotic biomarkers (Bax, caspase 3, PARP) and cellular–molecular damage biomarker (γH2AX) were significantly increased in kidney IR animals compared with sham controls. The findings of the present study therefore strengthen the findings of previous studies [11-14]. These parameters were significantly ameliorated by CsA or ADMSC monotherapy, lending support to previous studies that have also shown CsA or ADMSC had anti-apoptotic and anti-ROS/antioxidative stress capacities [5,6,21,23]. Distinctive to the current study was that co-treatment using CsA and ADMSC was superior to either monotherapy alone at ameliorating these mediators. These findings again partially explain why acute kidney IR injury was reduced further by our CsA and ADMSC co-treatment strategy. Surprisingly, the number of proliferating cell nuclear antigen-positive cells in the kidney, a sign of repair and regeneration, was found to be notably increased in the IR animals without treatment compared with those animals with treatment. We suggest that this could be explained as a result of a rigorous and struggling response to the IR injury.

In contrast to the findings of inflammatory, oxidative stress and ROS biomarkers, the antioxidant and anti-inflammatory biomarkers (HO-1, NQO 1, GPx, GR, IL-10, prostaglandin E2, eNOS) were notably lower in IR animals that were significantly reversed by either CsA or ADMSC treatment. The impact of ADMSC or CsA on enhancing the generation of antioxidant/anti-inflammatory mediators and hence protecting against organ damage from ischemia or IR have been emphasized by previous reports [5,6,21,23-25]; in this way, our findings were consistent with those of others. However, we demonstrated that combination therapy with CsA and ADMSCs contributed enhanced generation of these advantageous biomarkers, lending further explanation to why kidney IR injury was diminished more by co-treatment with CsA and ADMSC than mono-treatment with either.

Of interest, IF staining revealed significantly lower numbers of endothelial surface markers (that is, CD31^+^ and vWF^+^ cells) and EPC surface markers (that is, CXCR4^+^, SDF-1α^+^ cells) in IR animals being significantly preserved by CsA therapy, but being preserved significantly better by ADMSC therapy. One previous study has also demonstrated that autologous transfusion of ADMSCs to animals with acute IR-injured kidney enhanced angiogenesis in IR kidney tissue [5], supporting our findings. More importantly, combination therapy with CsA and ADMSC not only contributed to an increase in the numbers of endothelial cell and EPC surface markers but also to an increased number of small vessels (representing angiogenesis) in IR-injured kidney. These findings lend further explanation to why renal function was better preserved and kidney injury scores were further reduced in animals receiving co-treatment with CsA and ADMSC.

### Study limitations

**Figure 10:**
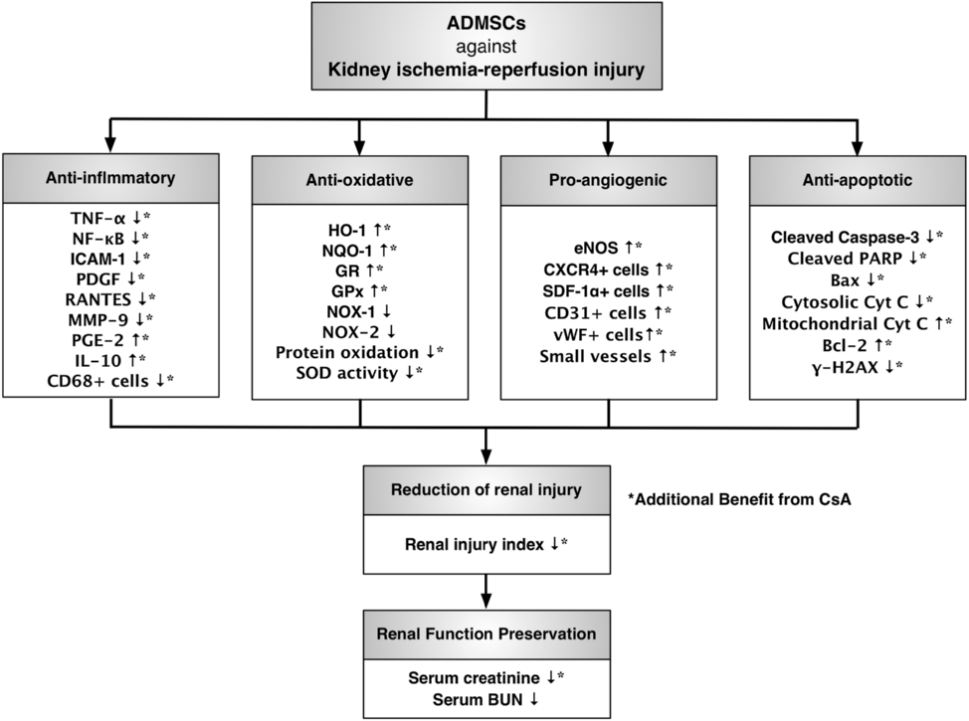
**Proposed mechanisms underlying therapeutic effects of cyclosporine, adipose-derived mesenchymal stem cells, and combination therapy.** Proposed mechanisms underlying therapeutic effects of cyclosporine (CsA), adipose-derived mesenchymal stem cells (ADMSCs), and combined CsA and ADMSCs on improving renal ischemia–reperfusion. ICAM-1, intercellular adhesion molecule-1; PDGF, platelet-derived growth factor; RANTES, regulated and normal T-cell expressed and secreted; MMP-9, matrix metalloproteinase; PGE2, prostaglandin E2; HO-1, heme oxygenase-1; NQO, NAD(P)H quinone oxidoreductase; GR, glutathione reductase; GPx, glutathione peroxidase; NOX, NAD(P)H oxidase; eNOS, endothelial nitric oxide synthase; SDF-1α, stromal cell-derived factor-1α; vWF, von Willebrand factor; PARP, cleaved poly(ADP-ribose) polymerase; BUN, blood urea nitrogen.

This study has limitations. First, although extensive biomarkers that play crucial roles in acute kidney IR injury were assayed, the precise signaling pathway(s) governing the therapeutic effects of CsA treatment, ADMSC treatment or CsA/ADMSC co-treatment have not been elucidated. We have, however, proposed mechanisms based on the findings of the current study, as summarized in Figure 10. In this way, we speculate that the mechanism of the enhanced protective effect of CsA-assisted ADMSC therapy was through augmentation of anti-inflammatory, antioxidative and anti-apoptotic effect, and probably also through an effect of CsA on enhancing retention of ADMSCs, which in turn facilitated the generation of angiogenesis factors. Second, without examining the impact of different regimens of CsA on the setting of acute kidney IR injury, we did not know whether the CsA dose used (20 mg/kg) in this study was a low dose, a high dose or just an optimal dose. Further investigation is recommended prior to application of this strategic management for transplant patients to prevent cyclosporine-related toxicity and complication, especially during the prolonged use of this drug.

## Conclusion

Our results strongly indicate that combination therapy using both CsA and ADMSCs provided superior protection against acute IR-induced kidney injury then either monotherapy alone. We highlight the clinical relevance of this co-treatment regimen for patients with acute kidney IR injury, especially in those experiencing rejection of their renal transplant. Furthermore, our results may provide important clinical relevance to the possibility of cyclosporine withdrawal in transplant patients by adding ADMSCs to mitigate complications of prolonged immunosuppressive therapy.

## Abbreviations

ADMSC: Adipose-derived mesenchymal stem cell; Bax: Bcl-2-associated X protein; BUN: Blood urea nitrogen; CsA: Cyclosporine A; CXCR4: C-X-C chemokine receptor type 4; DMEM: Dulbecco’s modified eagle’s medium; eNOS: Endothelial nitric oxide synthase; EPC: Endothelial progenitor cell; GPx: Glutathione peroxidase; GR: Glutathione reductase; H & E: Hematoxylin and eosin; HO-1: Heme oxygenase-1; IF: Immunofluorescent; IHC: Immunohistochemical; IL: Interleukin; IR: Ischemia–reperfusion; mAb: Monoclonal antibody; MPT: Mitochondrial permeability transition; MSC: Mesenchymal stem cell; NF-κB: Nuclear factor kappa-light-chain-enhancer of activated B cells; NOX: NADPH oxidase; NQO-1: NADPH dehydrogenase (quinone)-1; PARP: poly(ADP-ribose) polymerase; PCR: Polymerase chain reaction; RANTES: Regulated upon activation normal T-cell expressed and presumably secreted; ROS: Reactive oxygen species; SDF-1α: Stromal cell-derived factor-1α; SOD: Superoxide dismutase; TNF: Tumor necrosis factor; γH2AX: Phosphorylated H2A histone family member X.

## Competing interests

The authors declare that they have no competing interests.

## Authors’ contributions

Y-TC, C-CY, and C-KS carried out the animal surgery and sample collection. Y-YZ and J-LY carried out the molecular and biochemical analysis. C-LCha and J-JS performed the pathophysiological examination. C-LCho, SC, and T-HT participated in data acquisition and performed statistical analysis. CGW participated in data analysis and manuscript editing. SL and H-KY conceived of the study, participated in its design and coordination, and helped in drafting the manuscript. All authors read and approved the final manuscript.

## References

[B1] Parikh CR Coca SG Wang Y Masoudi FA Krumholz HM Long-term prognosis of acute kidney injury after acute myocardial infarction Arch Intern Med 2008 168 987 995 10.1001/archinte.168.9.987 18474763

[B2] Friedericksen DV Van der Merwe L Hattingh TL Nel DG Moosa MR Acute renal failure in the medical ICU still predictive of high mortality S Afr Med J 2009 99 873 875 20459997

[B3] Lameire N Van Biesen W Vanholder R Acute renal failure Lancet 2005 365 417 430 15680458 10.1016/S0140-6736(05)17831-3

[B4] Sementilli A Franco M Renal acute cellular rejection: correlation between the immunophenotype and cytokine expression of the inflammatory cells in acute glomerulitis, arterial intimitis, and tubulointerstitial nephritis Transplant Proc 2010 42 1671 1676 10.1016/j.transproceed.2009.11.042 20620497

[B5] Chen YT Sun CK Lin YC Chang LT Chen YL Tsai TH Chung SY Chua S Kao YH Yen CH Shao PL Chang KC Leu S Yip HK Adipose-derived mesenchymal stem cell protects kidneys against ischemia-reperfusion injury through suppressing oxidative stress and inflammatory reaction J Transl Med 2011 9 51 10.1186/1479-5876-9-51 21545725 PMC3112438

[B6] Sun CK Yen CH Lin YC Tsai TH Chang LT Kao YH Chua S Fu M Ko SF Leu S Yip HK Autologous transplantation of adipose-derived mesenchymal stem cells markedly reduced acute ischemia-reperfusion lung injury in a rodent model J Transl Med 2011 9 118 10.1186/1479-5876-9-118 21781312 PMC3155151

[B7] Yen CH Lin KC Leu S Sun CK Chang LT Chai HT Chung SY Chang HW Ko SF Chen YT Yip HK Chronic exposure to environmental contaminant nonylphenol exacerbates adenine-induced chronic renal insufficiency: role of signaling pathways and therapeutic impact of rosuvastatin Eur J Pharm Sci 2012 46 455 467 10.1016/j.ejps.2012.03.009 22484332

[B8] Thadhani R Pascual M Bonventre JV Acute renal failure N Engl J Med 1996 334 1448 1460 10.1056/NEJM199605303342207 8618585

[B9] Xue JL Daniels F Star RA Kimmel PL Eggers PW Molitoris BA Himmelfarb J Collins AJ Incidence and mortality of acute renal failure in Medicare beneficiaries, 1992 to 2001 J Am Soc Nephrol 2006 17 1135 1142 10.1681/ASN.2005060668 16495381

[B10] Ali T Khan I Simpson W Prescott G Townend J Smith W Macleod A Incidence and outcomes in acute kidney injury: a comprehensive population-based study J Am Soc Nephrol 2007 18 1292 1298 10.1681/ASN.2006070756 17314324

[B11] Chen Q Camara AK Stowe DF Hoppel CL Lesnefsky EJ Modulation of electron transport protects cardiac mitochondria and decreases myocardial injury during ischemia and reperfusion Am J Physiol Cell Physiol 2007 292 C137 C147 16971498 10.1152/ajpcell.00270.2006

[B12] Chen Q Moghaddas S Hoppel CL Lesnefsky EJ Ischemic defects in the electron transport chain increase the production of reactive oxygen species from isolated rat heart mitochondria Am J Physiol Cell Physiol 2008 294 C460 C466 18077608 10.1152/ajpcell.00211.2007

[B13] Hirayama A Nagase S Ueda A Oteki T Takada K Obara M Inoue M Yoh K Hirayama K Koyama A In vivo imaging of oxidative stress in ischemia–reperfusion renal injury using electron paramagnetic resonance Am J Physiol Renal Physiol 2005 288 F597 F603 10.1152/ajprenal.00020.2004 15536173

[B14] Nilakantan V Hilton G Maenpaa C Van Why SK Pieper GM Johnson CP Shames BD Favorable balance of anti-oxidant/pro-oxidant systems and ablated oxidative stress in Brown Norway rats in renal ischemia–reperfusion injury Mol Cell Biochem 2007 304 1 11 10.1007/s11010-007-9480-z 17458515

[B15] Halestrap AP Pasdois P The role of the mitochondrial permeability transition pore in heart disease Biochim Biophys Acta 2009 1787 1402 1415 10.1016/j.bbabio.2008.12.017 19168026

[B16] Javadov S Karmazyn M Escobales N Mitochondrial permeability transition pore opening as a promising therapeutic target in cardiac diseases J Pharmacol Exp Ther 2009 330 670 678 10.1124/jpet.109.153213 19509316

[B17] Morin D Assaly R Paradis S Berdeaux A Inhibition of mitochondrial membrane permeability as a putative pharmacological target for cardioprotection Curr Med Chem 2009 16 4382 4398 10.2174/092986709789712871 19835566 PMC2874726

[B18] Argaud L Gateau-Roesch O Muntean D Chalabreysse L Loufouat J Robert D Ovize M Specific inhibition of the mitochondrial permeability transition prevents lethal reperfusion injury J Mol Cell Cardiol 2005 38 367 374 10.1016/j.yjmcc.2004.12.001 15698843

[B19] Kim JS Jin Y Lemasters JJ Reactive oxygen species, but not Ca^2+^ overloading, trigger pH- and mitochondrial permeability transition-dependent death of adult rat myocytes after ischemia-reperfusion Am J Physiol Heart Circ Physiol 2006 290 H2024 H2034 10.1152/ajpheart.00683.2005 16399872

[B20] Argaud L Gateau-Roesch O Chalabreysse L Gomez L Loufouat J Thivolet-Bejui F Robert D Ovize M Preconditioning delays Ca^2+^−induced mitochondrial permeability transition Cardiovasc Res 2004 61 115 122 10.1016/j.cardiores.2003.11.003 14732208

[B21] Yuen CM Sun CK Lin YC Chang LT Kao YH Yen CH Chen YL Tsai TH Chua S Shao PL Leu S Yip HK Combination of cyclosporine and erythropoietin improves brain infarct size and neurological function in rats after ischemic stroke J Transl Med 2011 9 141 10.1186/1479-5876-9-141 21864394 PMC3177906

[B22] Piot C Croisille P Staat P Thibault H Rioufol G Mewton N Elbelghiti R Cung TT Bonnefoy E Angoulvant D Macia C Raczka F Sportouch C Gahide G Finet G André-Fouët X Revel D Kirkorian G Monassier JP Derumeaux G Ovize M Effect of cyclosporine on reperfusion injury in acute myocardial infarction N Engl J Med 2008 359 473 481 10.1056/NEJMoa071142 18669426

[B23] Sheu JJ Chua S Sun CK Chang LT Yen CH Wu CJ Fu M Yip HK Intra-coronary administration of cyclosporine limits infarct size, attenuates remodeling and preserves left ventricular function in porcine acute anterior infarction Int J Cardiol 2011 147 79 87 10.1016/j.ijcard.2009.08.008 19751953

[B24] Yip HK Chang LT Wu CJ Sheu JJ Youssef AA Pei SN Lee FY Sun CK Autologous bone marrow-derived mononuclear cell therapy prevents the damage of viable myocardium and improves rat heart function following acute anterior myocardial infarction Circ J 2008 72 1336 1345 10.1253/circj.72.1336 18654023

[B25] Leu S Sun CK Sheu JJ Chang LT Yuen CM Yen CH Chiang CH Ko SF Pei SN Chua S Youssef AA Wu CJ Yip HK Autologous bone marrow cell implantation attenuates left ventricular remodeling and improves heart function in porcine myocardial infarction: an echocardiographic, six-month angiographic, and molecular-cellular study Int J Cardiol 2011 150 156 168 10.1016/j.ijcard.2010.03.007 20466442

[B26] Assmus B Schachinger V Teupe C Britten M Lehmann R Dobert N Grunwald F Aicher A Urbich C Martin H Hoelzer D Dimmeler S Zeiher AM Transplantation of Progenitor Cells and Regeneration Enhancement in Acute Myocardial Infarction (TOPCARE-AMI) Circulation 2002 106 3009 3017 10.1161/01.CIR.0000043246.74879.CD 12473544

[B27] Dai W Hale SL Martin BJ Kuang JQ Dow JS Wold LE Kloner RA Allogeneic mesenchymal stem cell transplantation in postinfarcted rat myocardium: short- and long-term effects Circulation 2005 112 214 223 10.1161/CIRCULATIONAHA.104.527937 15998673

[B28] Le Blanc K Tammik L Sundberg B Haynesworth SE Ringden O Mesenchymal stem cells inhibit and stimulate mixed lymphocyte cultures and mitogenic responses independently of the major histocompatibility complex Scand J Immunol 2003 57 11 20 10.1046/j.1365-3083.2003.01176.x 12542793

[B29] Thum T Bauersachs J Poole-Wilson PA Volk HD Anker SD The dying stem cell hypothesis: immune modulation as a novel mechanism for progenitor cell therapy in cardiac muscle J Am Coll Cardiol 2005 46 1799 1802 10.1016/j.jacc.2005.07.053 16286162

[B30] Shi D Liao L Zhang B Liu R Dou X Li J Zhu X Yu L Chen D Zhao RC Human adipose tissue-derived mesenchymal stem cells facilitate the immunosuppressive effect of cyclosporin A on T lymphocytes through Jagged-1-mediated inhibition of NF-κB signaling Exp Hematol 2011 39 214 224 e211 10.1016/j.exphem.2010.10.009 21078360

[B31] Melnikov VY Faubel S Siegmund B Lucia MS Ljubanovic D Edelstein CL Neutrophil-independent mechanisms of caspase-1- and IL-18-mediated ischemic acute tubular necrosis in mice J Clin Invest 2002 110 1083 1091 12393844 10.1172/JCI15623 PMC150794

[B32] Sun CK Chang LT Sheu JJ Chiang CH Lee FY Wu CJ Chua S Fu M Yip HK Bone marrow-derived mononuclear cell therapy alleviates left ventricular remodeling and improves heart function in rat-dilated cardiomyopathy Crit Care Med 2009 37 1197 1205 10.1097/CCM.0b013e31819c0667 19242323

